# Neuroprotective Effects of Extracts from Tiger Milk Mushroom *Lignosus rhinocerus* Against Glutamate-Induced Toxicity in HT22 Hippocampal Neuronal Cells and Neurodegenerative Diseases in *Caenorhabditis elegans*

**DOI:** 10.3390/biology10010030

**Published:** 2021-01-05

**Authors:** Parinee Kittimongkolsuk, Nattaporn Pattarachotanant, Siriporn Chuchawankul, Michael Wink, Tewin Tencomnao

**Affiliations:** 1 Graduate Program in Clinical Biochemistry and Molecular Medicine, Department of Clinical Chemistry, Faculty of Allied Health Sciences, Chulalongkorn University, Bangkok 10330, Thailand; parinee.k@student.chula.ac.th; 2 Institute of Pharmacy and Molecular Biotechnology, Im Neuenheimer Feld 364, Heidelberg University, 69120 Heidelberg, Germany; 3 Department of Clinical Chemistry, Faculty of Allied Health Sciences, Chulalongkorn University, Bangkok 10330, Thailand; nat.ahs11@gmail.com; 4 Immunomodulation of Natural Products Research Group, Faculty of Allied Health Sciences, Chulalongkorn University, Bangkok 10330, Thailand; siriporn.ch@chula.ac.th; 5 Department of Transfusion Medicine and Clinical Microbiology, Faculty of Allied Health Sciences, Chulalongkorn University, Bangkok 10330, Thailand

**Keywords:** *Lignosus rhinocerus*, HT22, glutamate toxicity, neuroprotection, oxidative stress, *Caenorhabditis elegans*, chemotaxis, PolyQ40

## Abstract

**Simple Summary:**

Neurodegenerative diseases are recognized as one of the major public health issues in aging populations worldwide. High reactive oxygen species (ROS) cause oxidative stress, leading to cellular injury and neuronal cell death. While it has been used as traditional medicine, little is known about the neuroprotective effect of the Tiger Milk Mushroom *Lignosus rhinocerus* (LR). The aims of this study were to investigate the neuroprotective effect of three extracts of LR, including ethanol extract (LRE), cold water extract (LRC) and hot water extract (LRH), against glutamate-induced oxidative stress in mouse hippocampal (HT22) cells (in vitro model) as well as to determine their effect in *Caenorhabditis elegans* (in vivo model). We found that only LRE exhibited neuroprotective effects both in vitro (alleviation of glutamate-induced ROS in HT22 cells, resulting in increased cell survival) and in vivo (prevention of neurotoxicity in *C. elegans*). Therefore, active chemical constituents in LRE may serve as neuroprotectant candidates. Nevertheless, LRE extracts should be extensively studied for their neuroprotective activity in the future.

**Abstract:**

Despite the Tiger Milk Mushroom *Lignosus rhinocerus* (LR) having been used as a traditional medicine, little is known about the neuroprotective effects of LR extracts. This study aims to investigate the neuroprotective effect of three extracts of LR against glutamate-induced oxidative stress in mouse hippocampal (HT22) cells as well as to determine their effect in *Caenorhabditis elegans.* In vitro, we assessed the toxicity of three LR extracts (ethanol extract (LRE), cold-water extract (LRC) and hot-water extract (LRH)) and their protective activity by MTT assay, Annexin V-FITC/propidium iodide staining, Mitochondrial Membrane Potential (MMP) and intracellular ROS accumulation. Furthermore, we determined the expression of antioxidant genes (catalase (CAT), superoxide dismutase (SOD1 and SOD2) and glutathione peroxidase (GPx)) by qRT-PCR. In vivo, we investigated the neuroprotective effect of LRE, not only against an Aβ-induced deficit in chemotaxis behavior (Alzheimer model) but also against PolyQ40 formation (model for Morbus Huntington) in transgenic *C. elegans*. Only LRE significantly reduced both apoptosis and intracellular ROS levels and significantly increased the expression of antioxidant genes after glutamate-induced oxidative stress in HT22 cells. In addition, LRE significantly improved the Chemotaxis Index (CI) in *C. elegans* and significantly decreased PolyQ40 aggregation. Altogether, the LRE exhibited neuroprotective properties both in vitro and in vivo.

## 1. Introduction

Neurodegenerative disorders including Alzheimer’s diseases (AD), Parkinson’s disease (PD) and Huntington’s disease (HD) involve degeneration or death of nerve cells [1,2]. One of the factors that plays a complex role in these diseases is reactive oxygen species (ROS). Normally ROS are generated in the mitochondrial respiratory chain [3]. Excessive ROS induce oxidative stress causing lipid peroxidation that can lead to brain dysfunction and death [4]. In addition, ROS accumulation is detrimental for proteins, and nucleic acids (mutations), which may cause age-related diseases such as diabetes [5], and cancer [6,7]. Endogenous enzymes such as superoxide dismutase (SOD), catalase (CAT) and glutathione peroxidase (GPx) play an important role in detoxifying ROS [8].

The Tiger Milk Mushroom *Lignosus rhinocerus* (LR), found in Southeast Asia and China, has been used in folk medicine for asthma treatment [9]. Furthermore, this mushroom has anti-inflammatory, antioxidant, anti-proliferative, immuno-modulating and anti-HIV-1 activities in addition to a promotion of neurite outgrowth in PC-12 cells [10,11,12]. The bioactive compounds of LR consist of 1,3-β- and 1,6-β-glucans which are in a group of β-D-glucose polysaccharides, lectin which is a glycoprotein, laccase which is a copper-containing oxidase enzyme and other fungal immune-modulatory proteins (FIPs), and antioxidant proteins [13]. Besides carbohydrates and proteins, there are quinones, flavonoid-like compounds, cerebrosides, which are commonly known for a group of glycosphingolipids, and important components in animal muscle and nerve cell membranes, isoflavones, catechols, amines, triacylglycerols, sesquiterpenes and steroids [9,14].

Mouse hippocampal (HT22) cells were used to study glutamate-induced oxidative stress. Glutamate can enhance intracellular ROS levels and lead to oxidative stress in HT22 because of their lack of an ionotrophic glutamate receptor [15]. In consequence, the oxidative stress damages nerve cells and causes cell death.

*C. elegans*, a free-living nematode, contains many genes, which are homologues to those of humans [16]. This nematode has a short lifespan and is well suited for experiments. *C. elegans* is widely used as an animal model for the study of oxidative stress, aging, longevity and neurodegenerative diseases [17,18].

However, no or few studies have reported the protective effects of extracts from the Tiger Milk Mushroom in brain cells. Thus, in this study, we set out to investigate the potential neuroprotective effect of LR extracts against glutamate-induced oxidative stress, and also determined their effect on the expression of antioxidant genes in HT22 cells. In addition, we investigated potential neuroprotective effects of LR extracts in *C. elegans* as an in vivo model for Morbus Alzheimer and Huntington.

## 2. Materials and Methods

### 2.1. Chemicals and Reagents

Analytical grade ethanol was purchased from Merck (Darmatadt, Germany). Dimethyl sulfoxide (DMSO), 2’, 7’-dichlorodihydrofluorescein diacetate (H_2_DCFDA) from Sigma-Aldrich GmbH (Steinheim, Germany). Dulbecco’s modified Eagle medium (DMEM)/low glucose, fetal bovine serum (FBS) and penicillin-streptomycin solution (10,000 units/mL of penicillin and 10,000 g/mL of streptomycin) were purchased from HyClone (Logan, UT, USA). Chloroform, diethyl pyrocarbonate (DEPC), isopropanol and L-glutamic acid were from Sigma-Aldrich (St. Louis, MO, USA); primers and RT Premix were purchased from Bioneer (Daejeon, Korea). Phosphate Buffered Saline (PBS) was from Hyclone. Trypan Blue Stain and Trizol Reagent were from Invitrogen (Invitrogen, Carlsbad, CA, USA). Sodium azide was purchased from AppliChem GmbH (Darmstadt, Germany), and EGCG was purchased from Sigma–Aldrich (München, Germany).

### 2.2. Mushroom Extraction

A powder of cultivated strain TM02 of *L. rhinocerus* (LR) was obtained from LiGNO Biotech™ Sdn Bhd, Selangor, Malaysia. This powder was extracted into three fractions with ethanol, cold water and hot water using the maceration technique. Briefly, 100 g of LR powder was macerated with 1 L of ethanol and the extract was placed on the shaker at 4 °C for 24 h. After that, it was filtered by using Whatman® No.2 filter paper and ethanol was removed by rotary evaporation (Heidolph, Laborota 4011) to yield the crude ethanol extract (LRE). Cold water extraction: 100 g of LR powder was suspended in sterile water and placed on the shaker at 4 °C for 24 h. For hot water extraction, sclerotial powder was extracted with water at 95–100 °C for 2 h. After that, the mixture was filtered and freeze dried by lyophilizer (ModulyoD freeze dryer, Thermo Fisher Scientific, Waltham, MA, USA) to give a crude cold-water extract (LRC) and a crude hot-water extract (LRH). Yields of LRE, LRC and LRH were 0.73 g, 11.07 g and 10.13 g, respectively. Before starting the experiment, the crude extract of LRE was dissolved in DMSO, while the crude extracts of LRC and LRH were dissolved in sterile water to make the 100 mg/mL stock solution.

### 2.3. Cell Culture and Treatments

Mouse hippocampal HT22 cells (a generous gift from Professor David Schubert at the Salk Institute, San Diego, CA, USA) were cultured in a DMEM medium (Hyclone), supplied with 10% fetal bovine serum, in a humidified atmosphere containing 5% CO_2_ at 37 °C. For treatment, The HT22 cells were divided into 10 groups including control group; DMSO-treated group; three control extract groups (LRE-, LRC- and LRH-treated groups at the same concentration 25, 50, 100 and 200 µg/mL in all groups); 0.25 mM N-acetylcysteine (NAC) as a positive control; four co-treatment groups with 5 mM glutamate including three extract-treated groups (LRE-, LRC- and LRH-treated groups at the same concentration 25, 50, 100 and 200 µg/mL) and 0.25 mM NAC + 5 mM glutamate. All groups were incubated for 14 h in 5% CO_2_ in a 37 °C incubator.

### 2.4. 3-(4,5-Dimethylthiazol-2-yl)-2,5-Diphenyltetrazolium Bromide Tetrazolium (MTT) Assay

MTT assay, a colorimetric assay, was used to assess metabolic activity in the cells. Briefly, HT22 cells were seeded into 96-well plates and incubated overnight at 5% CO_2_ at 37 °C. Next, the cells were treated with the group of treatments as mentioned above and incubated at 5% CO_2_ at 37 °C for 14 h. After incubation, an MTT solution was added to each well and incubated for 3 h more in the incubator. After that, the insoluble formazan was dissolved with 10% SDS and incubated in the incubator overnight. The MTT product was measured at 570 nm using a microplate reader. The percent of cell viability was calculated by the following formular.

% Cell growth = [(Abs 570 nm of treated group–blank)/(Abs 570 nm of control–blank)] × 100

### 2.5. Assessment of Apoptosis by Annexin V-FITC/Propidium Iodide (PI) Staining Using Flow-Cytometry

A fluorescein isothiocyanate (FITC) conjugated form of Annexin V is used to detect apoptotic cells. HT22 cells (1 × 10^5^ cells) were seeded in a 12-well plate and incubated overnight at 5% CO_2_ at 37 °C. Next, the cells were treated with the group of treatments as mentioned before and incubated in the incubator for 14 h more. After incubation, cells were harvested, washed and stained with annexin V/PI solution for 15 min in the dark. Live and dead cells were determined by using a BD FACSCalibur™ flow cytometer (BD Bioscience, Heidelberg, Germany). Data were collected for groups of at least 10,000 cells and results are shown as the percentage of apoptotic cells.

### 2.6. Mitochondrial Membrane Potential (MMP) Assay

The MMP was determined by using a commercial kit (Cell Signaling, Danvers, MA, USA) including the cationic dye TMRE (tetramethylrhodamine ethyl ester perchlorate) and a mitochondrial membrane potential disruptor CCCP (carbonylcyanide 3-chlorophenylhydrazone) as a positive control for the test. TMRE, a cell membrane permeable fluorescent dye, was accumulated in intact mitochondria. Depolarized or inactive mitochondria exhibit decreased membrane potential, resulting in reduced TMRE accumulation. Briefly, cells were seeded in a 96-well plate and incubated overnight at 5% CO_2_ at 37 °C. Next, the cells were treated with the group of treatments as mentioned before, except cells of the CCCP group, which reached a final volume of 100 µL/well and the cells were incubated in the incubator for 14 h. After incubation, CCCP was added in the positive control group to get a final concentration of 50 µM, and then cells were incubated at 37 °C for 15 min. After that, a TMRE solution was added to each well to get a final concentration of 200 nM and the plate was placed in an incubator (37 °C and 5% CO_2_) for 20 min. Next, the solution was removed and the cells were washed with 1X PBS and then 100 µL/well 1X PBS was added to the plate. The samples were measured with a microplate reader at an excitation of about 550 nm and emission of about 580 nm.

### 2.7. Assessment of Intracellular ROS Accumulation

Intracellular ROS were determined using the CM-H2DCFDA (general oxidative stress indicator). After 14 h treatment, 10 μM of H2DCFDA was added to HT22 cells and incubated for 30 min at 37 °C, followed by washing three times with PBS. The fluorescence intensity (excitation = 485 nm; emission = 535 nm) was measured using an EnSpire® Multimode Plate Reader (Perkin–Elmer, Waltham, MA, USA) and the photographs were obtained using an Axio Observer A1 fluorescence microscope (Carl Zeiss, Jena, Germany).

### 2.8. RNA Isolation and Quantitative RT-PCR

In brief, total RNA was isolated from specific treatment cells using Trizol reagent). Using Accupower RT Premix (Bioneer), 1 μg of total RNA was converted to cDNA. Quantitative real-time PCR reaction was performed by using the Green Star PCR Master Mix where SYBR Green was included (Bioneer). Then, the specific genes CAT, SOD1, SOD2 and GPx were determined by the Exicycler Real Time Quantitative Thermal Block (Bioneer). The specific primers were previously reported by our research group [19]. The relative expression of each gene was normalized to the internal control gene (β-actin).

### 2.9. C. elegans Strains and Maintenance

Strains CL2355 (smg-1(cc546) dvIs50 (pCL45 (snb-1::Abeta 1–42:3’ UTR (long) + mtl-2::GFP) I), CL2122 (dvIs15 ((pPD30.38)unc-54(vector) + (pCL26)mtl-2::GFP)) and AM141 (rmls133 (unc-54::54p::Q40::YFP)) were obtained from Caenorhabditis Genetics Center (University of Minnesota, Minneapolis, MN, USA). All worms were maintained on a Nematode Growth Medium (NGM) agar containing *Escherichia coli* OP50 as a food source and kept at 16 °C, except for AM141 which were kept at 20 °C.

Concerning age-synchronized worms, the eggs were isolated by a bleaching reagent (5M NaOH and 5% NaOCl). They were then vortexed for 10 min and centrifuged for 40 s at 1300 rpm. Next, the supernatant was discarded and the eggs washed with sterile water twice. Then, as much water as possible was removed. An 8 mL M9 buffer was added to a 60 × 15 mm petri dish and the eggs were transferred into the dish and placed in a 20 °C incubator for 16 h. The eggs in the M9 buffer were hatched and remained at the L1 larvae stage.

### 2.10. Assessment of Neuroprotective Effects in C. elegans Model

#### 2.10.1. Chemotaxis Assay

Transgenic worms, including CL2355 with a pan-neuronal expression of the Human Abeta peptide, and CL2122, a control worm, were used. This assay was slightly modified from Wu Y. et al. [20]. Both the synchronized L1 worms CL2355 and 2122 were transferred to an S-medium containing *E. coli* OP 50 as a food source. Each strain of worms was divided into a control group, a DMSO-treated group and an LRE-treated group at concentrations of 50, 100 and 200 µg/mL, and 50 µg/mL EGCG (dissolved in DMSO) as a positive control. All worms were kept at 16 °C for 36 h, and then shifted to 23 °C and incubated for a further 36 h. Increased temperature is required for the pan-neuronal Aβ1–42 expression in the strain CL2355. After the incubation, the worms were washed three times with an M9 buffer to completely remove *E. coli* OP50. Finally, around 40 worms were placed in the center of a chemotaxis agar plate (94 mm). Before placing the worms there, 1 μL of the attractant odorant (0.1% benzaldehyde in 99.8% ethanol) together with 1 μL of 1 M sodium azide, used as a worm paralyser, were added to one side of the plate. On the opposite side, 1 μL of the control odorant (99.8% ethanol) along with 1 μL of 1 M sodium azide were added. Then, all plates were kept at 23 °C for 1 h. After that, the worms were counted from both sides and the chemotaxis index (CI) was calculated by the following formular:

CI = (Na − Nc)/Nt(1)

Na: Number of worms at the attractant position
Nc: Number of worms at the control position
Nt: Total number of worms on the plate

#### 2.10.2. Assessment of PolyQ40 Aggregation

The synchronized transgenic AM141, expressing PolyQ40::YFP as a reporter gene, were treated the same as with the chemotaxis assay, Then, the worms were incubated at 20 °C for 72 h. After the incubation period, the worms were mounted on a glass slide with a drop of 10 mM sodium azide for paralysis and images of at least thirty worms per group were collected. Fluorescent expression was detected by BIOREVO BZ-9000 fluorescence microscope (Keyence Deutschland GmbH, Neu-Isenburg, Germany) using 10 × objective lens at constant exposure time. The number in the PolyQ40 aggregation, located in the muscle cells of the worms, was counted manually. The mean ± SEM was analyzed for three independent replications.

### 2.11. Statistical Analysis

Data are presented as the mean of three independent experiments (the mean ± SEM) and analyzed with GraphPad Prism 6. Statistical comparison between the control and treatments was performed using one-way ANOVA following Bonferroni’s method (post-hoc). Lifespan data were determined by log-rank (Mantel-Cox) tests followed by the Gehan–Breslow–Wilcoxon test. All the experiments were performed at least three times. Differences with *p* < 0.05 were considered statistically significant.

## 3. Results

### 3.1. Effect of LR Extracts Against Glutamate-Induced Cytotoxicity

**Figure 1:**
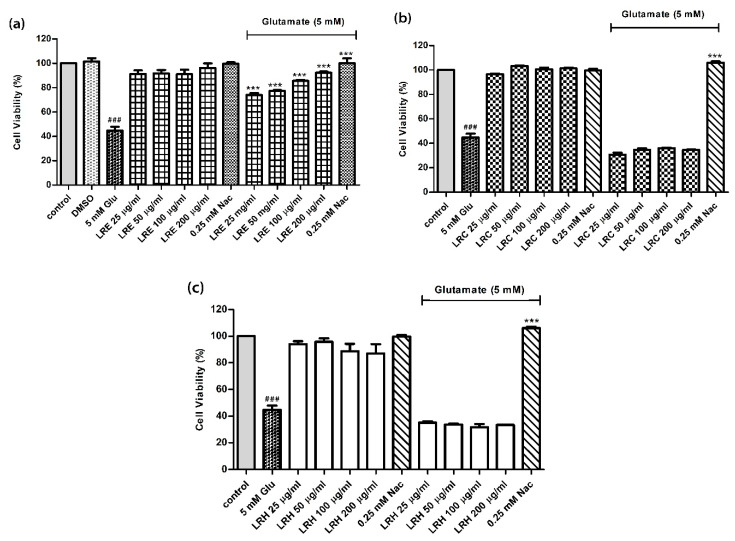
The protective effect of different concentrations of *Lignosus rhinocerus* (LR) extracts against glutamate-induced toxicity in HT22 cells. Viability of untreated control cells was set at 100%. (**a**) Treatment of cells with an LR ethanol extract (LRE) and LRE plus glutamate; (**b**) Treatment of cells with LRC and LRC plus glutamate; (**c**) Treatment of cells with LRH and LRH plus glutamate. Values are mean ± SEM of at least three independent runs. Significant differences: ^###^ *p* < 0.001 vs. control; *** *p* < 0.001 vs. glutamate alone.

The MTT assay was used to evaluate potential cell cytotoxicity of three extracts from LR—an ethanol extract (LRE), a cold-water extract (LRC) and a hot-water extract (LRH). The results showed that 5 mM glutamate significantly reduced the cell viability of HT22 cells by 44.71 ± 3.15% (*p* < 0.001 compared to control) (Figure 1). In addition, the viability of cells, treated with LRE, LRH and LRC at concentrations up to 200 µg/mL and 0.25 mM N-acetylcysteine (NAC), did not differ from that of the control cells. Moreover, co-treatment cells with 25, 50, 100 and 200 µg/mL of LRE and 5 mM glutamate revealed significant protective effects of LRE against cell death in a dose-dependent manner compared to glutamate-treated cells. Likewise, co-treatment with 0.25 NAC and 5 mM glutamate significantly increased cell viability to 94.67 ± 1.76% compared to glutamate-treated cells (Figure 1a). However, LRH and LRC had no effect (Figure 1b,c).

### 3.2. Anti-Apoptotic Activity of LR Extracts

**Figure 2:**
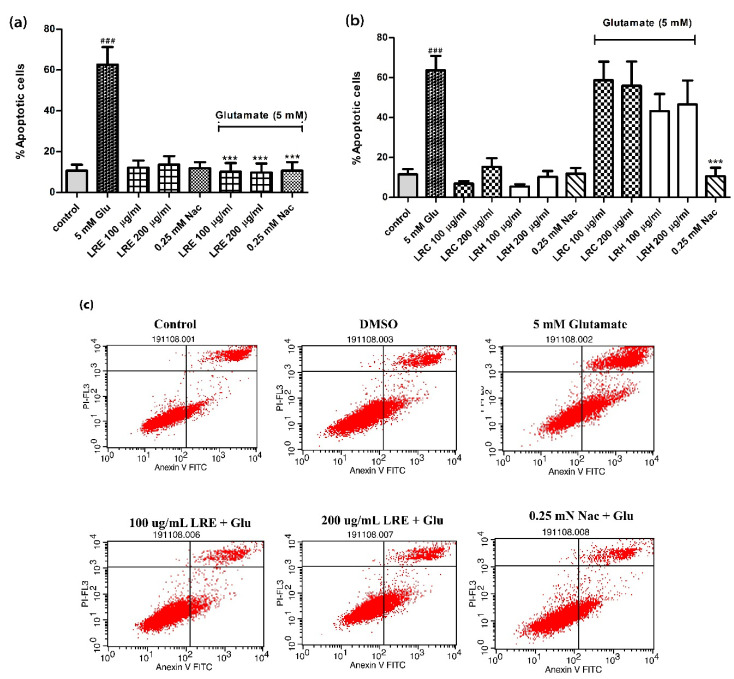
Quantitative flow cytometric analysis of the abundance of apoptotic HT22 cells using Annexin V-FITC/PI staining. Annexin V+/PI– cells are seen in the lower right are in the early stage and annexin V+/PI+ in the upper right quadrant are late stage. (**a**) Treatment of cells with glutamate, NAC and LRE alone or in combination with 5 mM glutamate; (**b**) Treatment of cells with glutamate, NAC and LRC and LRH alone or in combination with 5 mM glutamate; (**c**) Representative scatter plots of the distribution of annexin V and PI-stained cells. Values are mean ± SEM of at least three independent runs. ^###^ *p* < 0.001 vs. control; *** *p* < 0.001 vs. glutamate alone.

In a second step, we investigated the role of apoptosis in glutamate-induced cell death in HT22 cells. In accordance with the MTT results, we selected concentrations of 100 and 200 µg/mL of LR and 5 mM glutamate. We found that 5 mM glutamate increased the percentage of apoptotic cells from 10.71 ± 2.91% in controls to 62.64 ± 8.64%. Apoptosis in LRE- and NAC-treated cells was similar to that of controls. However, co-treatment cells with LRE and glutamate reduced the percentage of apoptotic cells to 10.18 ± 4.266% and 9.796 ± 4.416% (LRE) and 10.73 ± 4.175% (NAC) (Figure 2a). However, both LRC and LRH had no effects (Figure 2b).

### 3.3. Effect of LR Extracts on Mitochondrial Membrane Potential (MMP)

**Figure 3:**
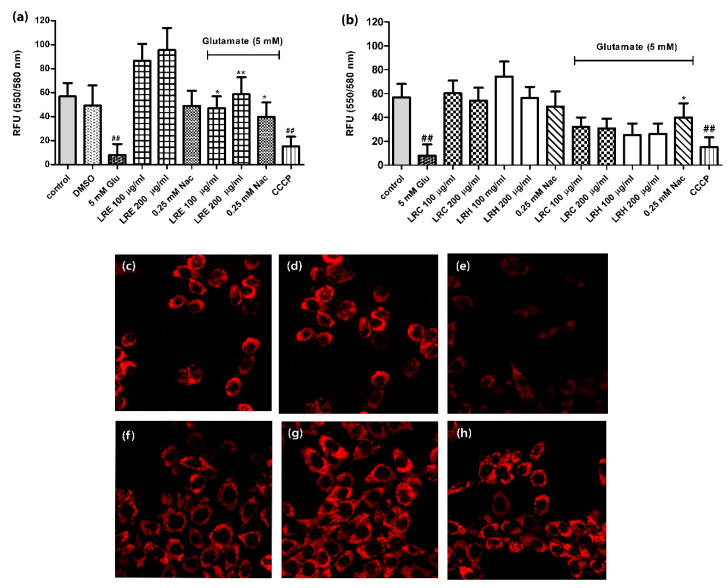
Protective effect of different concentrations of LR extracts on Mitochondrial Membrane Potential (MMP) in HT22 cells. (**a**) Treatment of cells with LRE, N-acetylcysteine (NAC), carbonylcyanide 3-chlorophenylhydrazone (CCCP) and LRE, NAC plus glutamate; (**b**) Treatment of cells with LRH, LRC and LRH, LRC plus glutamate; (**c**–**h**) Representative fluorescence micrographs with TMRE staining using fluorescence microscopy; (**c**) control group; (**d**) DMSO group; (**e**) 5 mM glutamate group; (**f**) 100 µg/mL LRE co-treatment group; (**g**) 200 µg/mL; (**h**) 0.25 NAC group. Values are mean ± SEM of at least three independent runs. ^##^ *p* < 0.01 vs. control; * *p* < 0.05; ** *p* < 0.01 vs. glutamate alone.

Glutamate reduced the MMP of HT22 cells to 8.00 ± 9.23 compared to control (56.81 ± 11.28). Similarly, the cells, treated with CCCP as a positive control for MMP, significantly decreased the fluorescence intensity to 15.29 ± 7.947. LRE and 0.25 mM NAC alone had no influence on MMP. However, a co-treatment of the cells with 100 and 200 μg/mL of LRE and 5 mM glutamate significantly improved the TMRE fluorescence intensity in a dose-dependent manner compared to glutamate-treated cells at 47.19 ± 9.774, and 58.62 ± 14.36, respectively. Moreover, the co-treatment with NAC had a positive effect (39.95 ± 11.89 (Figure 3a). The co-treatment of cells with LRC or LRH (100, and 200 μg/mL) with 5 mM glutamate showed low fluorescence intensity of TMRE but were not significantly different from 5 mM glutamate-treated cells (Figure 3b).

### 3.4. Effect of LR Extracts on Intracellular ROS Level

**Figure 4:**
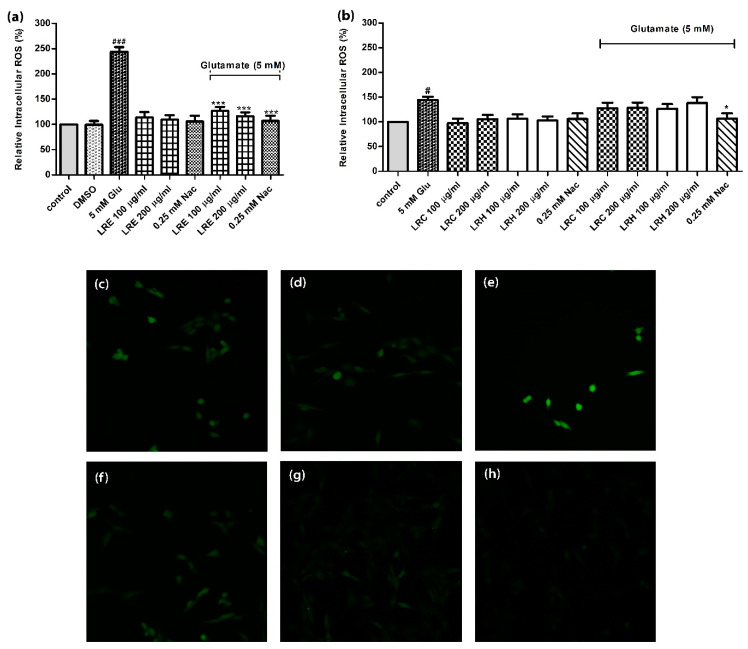
The effect of different concentrations of LR extracts on intracellular ROS accumulation in HT22 cells. ROS levels of untreated control cells were set at 100%. (**a**) Treatment of cells with glutamate, NAC and LRE alone or in combination with 5 mM glutamate; (**b**) Treatment of cells with glutamate, NAC and LRC and LRH alone or in combination with 5 mM glutamate; (**c**) Representative fluorescence micrographs of DCFH-DA stained control cells; (**d**) DMSO group; (**e**) 5 mM glutamate group; (**f**) 100 µg/mL LRE and (**g**) 200 µg/mL of LRE co-treatment groups; (**h**) 0.25 NAC group. Values are mean ± SEM of at least three independent experiments. ^#^ *p* < 0.05, ^###^ *p* < 0.001 vs. control; * *p* < 0.05; *** *p* < 0.001 vs. glutamate alone.

When HT22 cells were treated with 5 mM of glutamate alone, ROS concentrations increased significantly to 243.9 ± 9.22% (compared to control = 100%). Whereas, co-treatment of the cells with LRE (100 and 200 μg/mL) and 5 mM glutamate significantly reduced intracellular ROS accumulation in a dose-dependent manner compared to control to 126.6 ± 8.39%, and 116.1 ± 7.99% (Figure 4a); moreover, 0.25 mM NAC could counteract ROS levels (106.9 ± 10.33%) (Figure 4a). In contrast, LRC or LRH had no effects (Figure 4b).

### 3.5. Effect of LR extracts on Antioxidant Gene Expression

To better understand the antioxidant effects of LRE in previous experiments, we analysed the expression of genes that are potentially relevant in the antioxidant response of cells, such as the genes for catalase (CAT), superoxide dismutase (SOD1 and SOD2) and glutathione peroxidase (GPx).

**Figure 5:**
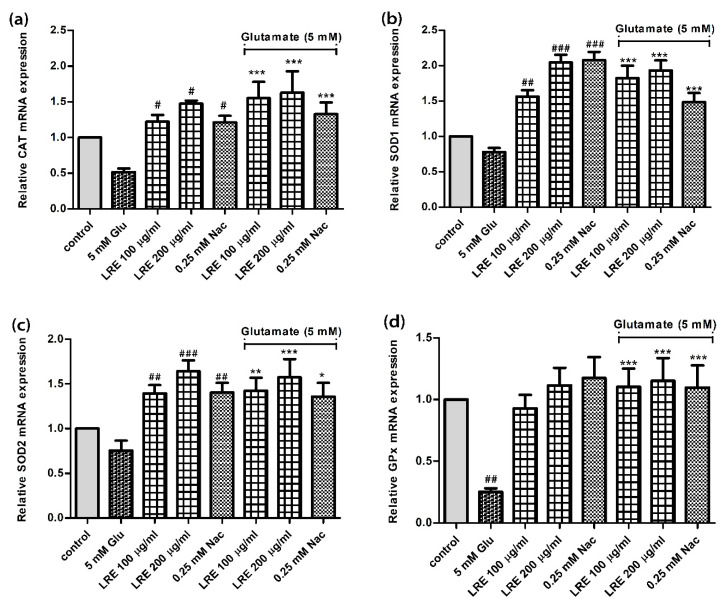
The effect of different concentrations of LRE extracts on the expression of antioxidant genes (catalase (CAT), superoxide dismutase (SOD1 and SOD2) and glutathione peroxidase (GPx)) in HT22 cells given alone or in combination with 5 mM glutamate. (**a**) CAT; (**b**) DOD1; (**c**) SOD2; (**d**) GPx. Values are mean ± SEM of at least three independent runs. ^#^ *p* < 0.05; ^##^ *p* < 0.01; ^###^ *p* < 0.001 vs. control; * *p* < 0.05; ** *p* < 0.01; *** *p* < 0.001 vs. glutamate alone.

Glutamate treatment reduced the expression of CAT and GPx in cells. The expression of CAT, SOD1, SOD2 and GPx was significantly enhanced in cells co-treated with 100 and 200 μg/mL LRE or NAC and 5 mM glutamate (Figure 5a–d).

### 3.6. Neuroprotective Effect of LR Extracts Against Aβ-Induced Deficit in Chemotaxis Behavior in C. elegans

The transgenic CL2355 worms express Aβ in nerve cells (Alzheimer model). As a physiological response, these nematodes show a reduced ability for chemotaxis. In order to determine potential neuroprotective effects, we selected three concentrations of LRE (50, 100 and 200 μg/mL) that gave good effects in HT22 cells.

**Figure 6:**
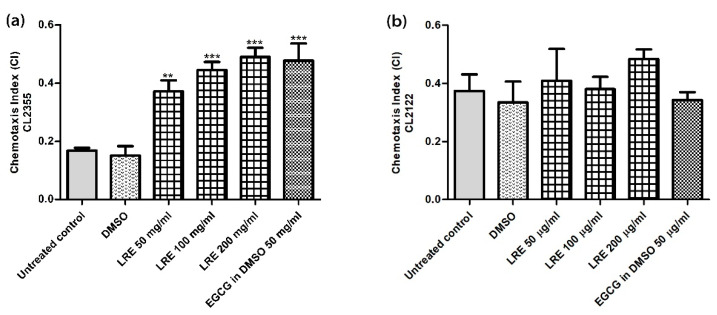
The effect of different concentrations of LRE extracts against *Aβ*-induced reduction of chemotaxis behavior in *C. elegans*. (**a**) treatment of CL2355 worms (*Aβ+*) with LRE and EGCG (positive control); (**b**) treatment of CL2122 worms (*Aβ-*) with LRE and EGCG (positive control). Values are mean ± SEM of at least three independent runs. ** *p* < 0.01; *** *p* < 0.001 vs. DMSO.

The Chemotaxis Index (CI) increased in a dose-dependent manner when treated with 50, 100 and 200 μg/mL LRE at 0.37 ± 0.04, 0.45 ± 0.03 and 0.49 ± 0.03, respectively, compared to 0.48 ± 0.06 in the EGCG positive control. However, there was no difference detected in CI in transgenic CL2122 worms (without Aβ) when treated with LRE and 50 μg/mL EGCG (Figure 6).

### 3.7. Neuroprotective Effect of LR Extracts on PolyQ40 Aggregation

**Figure 7:**
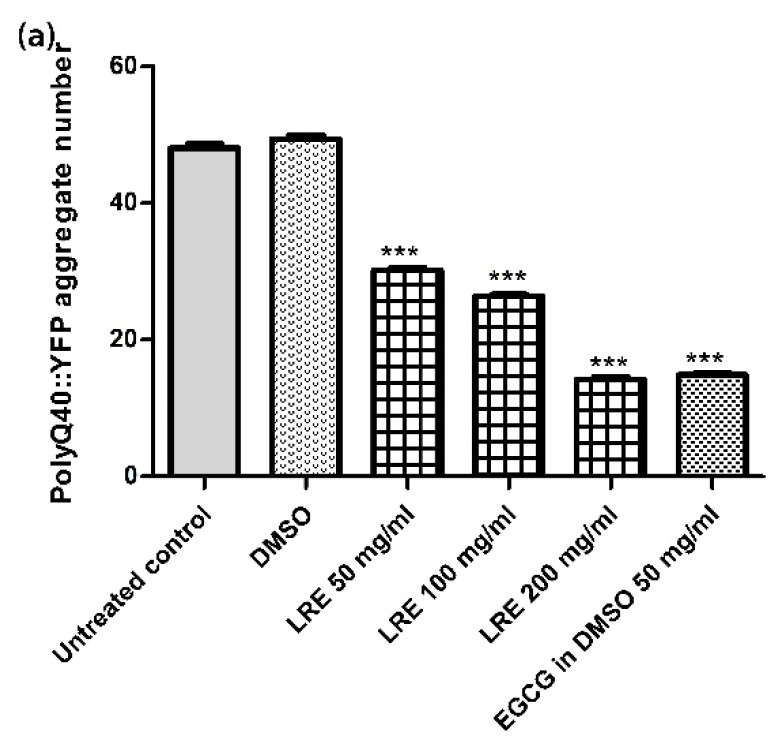

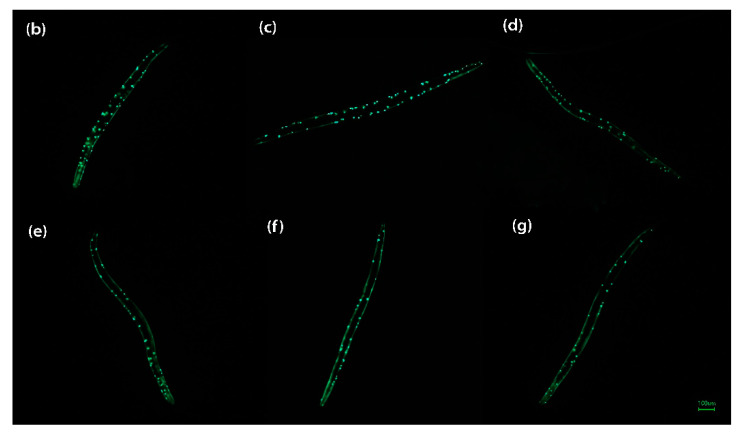
The effect of different concentrations of LRE extracts against PolyQ40 aggregation in *C. elegans*. (**a**) PolyQ40 formation after treatment with of different concentration of LRE; (**b**) Visualization of PolyQ40 formation by fluorescence microscopy: (**b**) untreated worms; (**c**) DMSO-treated worms; (**d**–**f**) after treatment with 50, 100 and 200 μg/mL of LRE; (**g**) worms treated with 50 μg/mL of EGCG. Values are mean ± SEM of at least three independent runs. *** *p* < 0.001 vs. DMSO.

The accumulation of PolyQ40 proteins in transgenic AM141 worms was used as a model for Huntigton. When AM141 worms were treated with 50, 100 and 200 μg/mL LRE, the number of PolyQ40 aggregates was reduced in a dose-dependent manner to 30.18 ± 0.41, 26.38 ± 0.28 and 14.21 ± 0.36, respectively, compared to the DMSO control (49.40 ± 0.65). Similarly, the worms treated with 50 μg/mL EGCG showed a significantly reduced PolyQ40 aggregate accumulation (Figure 7).

## 4. Discussion

Neurodegenerative disorders including Alzheimer’s disease (AD), Parkinson’s disease (PD) and Huntington’s Disease (HD) are related to an increase of intracellular reactive oxygen species (ROS) accumulation [21,22,23,24,25]. The antioxidants are the first defense to detoxify ROS. Recently, plants or natural products have been widely studied because most of them contain phytochemical constituents that are involved in antioxidant activities that have safe and minimal side effects [26,27,28,29]. *L. rhinocerus* (LR) or Tiger Milk Mushroom have been used as a folk medicinal mushroom for treatment of asthma [9]. Several studies have found that the cultivated Tiger Milk Mushroom sclerotia (TM02) performs better and has a higher content of bioactive compounds than the wild type [11,30]. They found carbohydrates, proteins and antioxidant proteins [13]. In addition, there are quinones and flavonoid-like compounds, acting as an antioxidant [14].

Glutamate, an excitatory neurotransmitter, has positive effects on several brain functions such as cognition, memory and learning [31]. However, excess glutamate leads to glutamate toxicity, and causes neuronal apoptosis [15]. There are two pathways for glutamate toxicity, including receptor-initiated excitotoxicity [32,33] and nonreceptor-mediated oxidative glutamate toxicity [34]. The latter is linked to glutamate-induced toxicity in HT22 cells because this immortalized cell line lacks the ionotrophic glutamate receptor. High concentrations of extracellular glutamate (>200 µM) cause glutamate-mediated oxidative stress by preventing cysteine uptake into cells through a glutamate/cysteine antiporter followed by depletion of intracellular cysteine, resulting in a glutathione reduction [35], which leads to ROS accumulation. Excessive ROS could damage cells and intracellular organelles such as mitochondria and endoplasmic reticulum (ER) in several ways. For example, ROS interacts with mitochondrial membranes, which leads to lipid peroxidation and membrane destabilization [36]. These processes alter mitochondrial membrane potential (MMP), which is a hallmark of mitochondrial dysfunction. MMP is a good target for assessing in vitro toxicity. A decrease in MMP and inactive mitochondria may be linked to apoptosis and associated with various diseases including cancer, neurodegenerative disorder and diabetes [37,38,39,40,41]. As previously reported, glutamate-induced apoptosis is mediated via the caspase-independent pathway in HT22 cells [42].

In the present study, we report, for the first time to our knowledge, the neuroprotection of LR extracts against glutamate-induced oxidative stress in HT22 cells and neurotoxicity in *C. elegans*. In this cell line, we induced oxidative stress by using 5 mM glutamate which has been reported to reduce approximately 50% of the cells [19,43]. Interestingly, we found that only LRE prevented cells from undergoing apoptosis after cotreatment by using Annexin V/PI straining that interacts strongly and specifically with exposed phosphatidylserine (PS), the marker of apoptosis [44]. The protective effect of LRE correlated with increasing MMP, antioxidant gene expressions (CAT, SOD1, SOD2 and GPx) and decreasing intracellular ROS accumulation. Studies on the chemical constituents of LR indicated that bioactive compounds from ethanol extracts performed better than aqueous extractions because the ethanol extract contained high levels of phenolic compounds and phospholipids such as linoleic, oleic and palmitic acid which acted as the antioxidants, whereas aqueous extracts contained a high proportion of polysaccharides, β-glucan and water-soluble components [45,46,47,48]. Some findings reported that aqueous extracts of LR showed antioxidant properties in vitro including DPPH and ABTS scavenging assay and contained phenolic compounds [9,13,30]. However, several findings found that mushroom β-glucan from the aqueous extract is involved in modulating the immune system and in anti-inflammatory, anti-cancer and antiviral activity [49,50,51]. Unlike β-glucan in mushrooms, β-glucan in barley showed a higher free radical scavenging property than in oats and yeast [52]. In addition, β-glucan in oats exert indirect antioxidant effects on immune cells [53]. Therefore, it could be possible that glucan in LRC and LRH are unlikely to be the major component that was responsible for protecting HT22 cells from glutamate-induced oxidative stress. Moreover, another possibility was the difference in yields of the extracts in our study. The yield of the LRE was 10 times lower than that of the LRC and LRH despite all extracts being applied at a similar concentration. Thus, the active ingredients in aqueous extracts may be lower than those in ethanolic extracts.

Furthermore, LRE exerted a neuroprotective effect against Aβ-induced deficits in chemotaxis behavior in *C. elegans*, leading to an increase in the CI of the CL 2355 transgenic strain, containing the human Aβ peptide that is also known as a hallmark of AD [54]. Similarly, LRE also induced a decrease in PolyQ40 aggregation that is related to the neurodegenerative diseases including Huntington’s disease and other polyglutamine diseases [55]. Our findings agree with several previous studies about phenolic compounds that have been found in plants or natural products and reported their effectiveness in preventing many diseases including neurodegenerative diseases [19,43,56,57,58,59,60,61,62,63,64,65,66,67]. Only LRE exerted the neuroprotective effect both in vitro and in vivo. Our findings suggested that LRE may be a new candidate for neurodegeneration protection, However, LRE extracts should be extensively studied for their neuroprotective activity.

## 5. Conclusions

From three extracts of the Tiger Milk Mushroom, only LRE showed the neuroprotective effect in hippocampal neuronal cells which is apparently mediated via inhibition of intracellular ROS accumulation and increases in both MMP and expression of antioxidant genes. In addition, LRE also had a neuroprotective effect in *C. elegans* in an Alzheimer and a Huntington model. However, further studies to identify the bioactive components and exact mechanism of LRE are required to support their ability for the development of a neuroprotective supplement in the future.

## Abbreviations

Aβ	amyloid beta
AD	Alzheimer’s disease
CAT	catalase
CI	chemotaxis index
DCFH2-DA	2’,7’-dichlorofluorescein diacetate;
EGCG	Epigallocatechin gallate
Glu	glutamate
GPx	glutathione peroxidase
LR	*Lignosus rhinocerus*
LRC	cold water extract of *L. rhinocerus*
LRE	ethanol extract of *L. rhinocerus*
LRH	hot water extract of *L. rhinocerus*
MMP	mitochondrial membrane potential
MTT	3-(4,5-dimethylthiazol-2-yl)-2,5-diphenyltetrazolium bromide
NAC	N-acetylcysteine
qRT-PCR	real time RT-PCR
ROS	reactive oxygen species
SOD	superoxide dismutase
